# Spinal deformity surgery is accompanied by serious complications: report from the Morbidity and Mortality Database of the Scoliosis Research Society from 2013 to 2020

**DOI:** 10.1007/s43390-022-00548-y

**Published:** 2022-07-15

**Authors:** Louis J. Bivona, John France, Conor S. Daly-Seiler, Douglas C. Burton, Lori A. Dolan, J. Justin Seale, Marinus de Kleuver, Emmanuelle Ferrero, David P. Gurd, Deniz Konya, William F. Lavelle, Vishal Sarwahi, Sanjeev J. Suratwala, Caglar Yilgor, Ying Li

**Affiliations:** 1 Department of Orthopaedic Surgery, West Virginia University School of Medicine, 1 Medical Center Dr, Morgantown, WV 26506 USA; 2 Department of Orthopaedic Surgery, University of Michigan, Ann Arbor, MI USA; 3 Department of Orthopaedic Surgery, University of Kansas Medical Center, Kansas City, KS USA; 4 Department of Orthopaedics and Rehabilitation, University of Iowa, Iowa City, IA USA; 5 Baptist Health, OrthoArkansas, Little Rock, AR USA; 6 Department of Orthopaedic Surgery, Sint Maartenskliniek, Radboud University Medical Center, Nijmegen, The Netherlands; 7 Department of Pediatric Orthopaedics, Robert-Debre Hospital, Paris, France; 8 Department of Orthopaedic Surgery, Cleveland Clinic, Cleveland, OH USA; 9 Department of Neurosurgery, Bahcesehir University School of Medicine, Istanbul, Turkey; 10 Department of Orthopedic Surgery, SUNY Upstate Medical University, Syracuse, NY USA; 11 Department of Pediatric Orthopaedics, Northwell Health System, New Hyde Park, NY USA; 12 New York Orthopaedic and Spine Center, Northwell Health System, New Hyde Park, NY USA; 13 Department of Orthopedics and Traumatology, Acibadem University School of Medicine, Istanbul, Turkey

**Keywords:** Deformity, Complications, Scoliosis

## Abstract

**Purpose:**

The Morbidity and Mortality (M&M) report of the Scoliosis Research Society (SRS) has been collected since 1965 and since 1968 submission of complications has been required of all members. Since 2009, the SRS has collected information on death, blindness, and neurological deficit, with acute infection being added in 2012 and unintentional return to the operating room (OR) being added in 2017. In this report, we use the most recent data submitted to the SRS M&M database to determine the rate of neurological deficit, blindness, acute infection, unintentional return to the OR, and death, while also comparing this information to previous reports.

**Methods:**

The SRS M&M database was queried for all cases from 2013 to 2020. The rates of death, vision loss, neurological deficit, acute infection, and unintentional return to the OR were then calculated and analyzed. The rates were compared to previously published data if available. Differences in complication rates between years were analyzed with Poisson regression with significance set at *α* = 0.05.

**Results:**

The total number of cases submitted per year varied with a maximum of 49,615 in 2018 and a minimum of 40,464 in 2020. The overall reported complication rate from 2013 to 2020 was 2.86%. The overall mortality rate ranged from 0.09% in 2018 to 0.14% in 2015. The number of patients with visual impairment ranged from 4 to 13 between 2013 and 2015 (no data on visual impairment were collected after 2015). The overall infection rate varied from 0.95 in 2020 to 1.30% in 2015. When the infection rate was analyzed based on spinal deformity group, the neuromuscular scoliosis group consistently had the highest infection rate ranging from 3.24 to 3.94%. The overall neurological deficit rate ranged from 0.74 to 0.94%, with the congenital kyphosis and dysplastic spondylolisthesis groups having the highest rates. The rates of unintentional return to the OR ranged from 1.60 to 1.79%. Multiple groups showed a statistically significant decreasing trend for infection, return to the operating room, neurologic deficit, and death.

**Conclusions:**

Neuromuscular scoliosis had the highest infection rate among all spinal deformity groups. Congenital kyphosis and dysplastic spondylolisthesis had the highest rate of neurological deficit postoperatively. This is similar to previously published data. Contrary to previous reports, neuromuscular scoliosis did not have the highest annual death rate. Multiple groups showed a statistically significant decreasing trend in complication rates during the reporting period, with only mortality in degenerative spondylolisthesis significantly trending upwards.

**Level of evidence:**

Level III.

**Supplementary Information:**

The online version contains supplementary material available at 10.1007/s43390-022-00548-y.

## Introduction

The Scoliosis Research Society (SRS) Morbidity and Mortality (M&M) database is an invaluable tool as it contains complications from a wide variety of procedures performed at a large number of institutions globally by mostly fellowship-trained spine surgeons. Since 2009, the SRS has collected information on death, blindness, and neurological deficit, with acute infection being added in 2012 and unintentional return to the operating room (OR) being added in 2017**.**

With the database containing a large number of cases from around the world, rates of rare events such as death and new neurological deficit can be determined over time in a real world setting. Ultimately, this allows those performing these procedures to better counsel patients on the risks and benefits of surgery.

A multitude of reports have been generated from the data collected by the SRS [1–11]. These studies include information regarding surgical complications for various pathologies, including adolescent idiopathic scoliosis, Scheuermann’s kyphosis, and thoracolumbar fixed sagittal plane deformity. Others have used the database to report on the complications of specific, frequently performed spinal procedures. In 2016, Burton et al. reported on the complications of the SRS M&M database from 2009 to 2012 [4]. In that time period, the authors found the overall mortality rate to range from 0.07 to 0.12%. Infection cases were first collected in the database in 2012 and the overall rate was 1.14%, with the highest rate occurring in patients with neuromuscular (NM) scoliosis (2.97%). Patients who had dysplastic spondylolisthesis and congenital kyphosis had the highest neurological deficit rates.

In this report, we use the most recent data submitted to the SRS M&M database to determine the rate of neurological deficit, blindness, acute infection, unintentional return to the OR, and death while also comparing this information to previous reports.

## Materials and methods

Reporting on complications related to spinal deformity procedures is an annual requirement for membership of the SRS. Members may opt out of reporting complications by paying a $300 fee, with approximately 70% of members submitting their complete data. Submitted cases are classified into specific spinal deformity groups (Table 1). The total number of cases is derived by summing the cases in each group.

**Table 1:** List of spinal deformity groups

List of spinal deformity groups in the SRS database
IS < 10
IS 10–18
IS > 18
Congenital scoliosis
Neuromuscular scoliosis
Other scoliosis
Isthmic spondylolisthesis
Degenerative spondylolisthesis
Dysplastic spondylolisthesis
Congenital kyphosis
Scheuermann’s kyphosis
Other kyphosis

Primary complications recorded include death (within 21 days of surgery), neurological deficit, acute infection (within 90 days of surgery), vision change, and unintentional return to the OR (within 90 days of the index surgery). Information collected on vision change was halted after 2015 due to the consistently low number of patients with this complication each year. Data on acute infection were first reported in 2012 and data on unintentional return to the OR were first reported in 2017.

Overall complication rates and complication rates for each spinal deformity group were calculated. Statistical analysis was performed using R 4.1.0. Poisson regression with significance set at *α* = 0.05 was used to model how each tracked complication rate changed between years for each spinal deformity group. Expected decrease and 95% confidence intervals were reported for models with *p* values below 0.05.

## Results

The total number of cases reported between 2013 and 2020 ranged from 40,464 in 2020 to 49,615 in 2018 (Tables 2, 3, 4, 5, 6). In all, 375,917 cases were submitted during that timeframe. The overall mortality rate ranged from 0.09% in 2018 to 0.14% in 2015. The highest group mortality rate was the other kyphosis group at 0.5%. These numbers generally align with what has been previously published [4]. From 2009 to 2012, the overall death rate ranged from 0.07 to 0.12%.

**Table 2:** Deaths

	2013 *N* (%)	2014 *N* (%)	2015 *N* (%)	2016 *N* (%)	2017 *N* (%)	2018 *N* (%)	2019 *N* (%)	2020 *N* (%)	*p* value
IS < 10	0 (0.00)	0 (0.00)	0 (0.00)	0 (0.00)	0 (0.00)	0 (0.00)	0 (0.00)	0 (0.00)	–
IS 10–18	0 (0.00)	1 (0.01)	3 (0.02)	1 (0.01)	3 (0.03)	3 (0.03)	1 (< 0.01)	0 (0.00)	0.85
IS > 18	4 (0.10)	5 (0.14)	6 (0.15)	5 (0.16)	4 (0.10)	4 (0.11)	3 (0.08)	10 (0.29)	0.29
Congenital scoliosis	2 (0.07)	3 (0.13)	2 (0.08)	0 (0.00)	1 (0.04)	3 (0.12)	3 (0.13)	0 (0.00)	0.71
Neuromuscular scoliosis	2 (0.05)	8 (0.22)	13 (0.34)	10 (0.29)	15 (0.39)	7 (0.19)	10 (0.30)	7 (0.25)	0.17
Other scoliosis	14 (0.24)	14 (0.24)	14 (0.24)	11 (0.22)	9 (0.16)	6 (0.10)	12 (0.20)	11 (0.24)	0.31
Isthmic spondylolisthesis	3 (0.08)	1 (0.03)	0 (0.00)	0 (0.00)	1 (0.03)	0 (0.00)	0 (0.00)	2 (0.07)	0.40
Degenerative spondylolisthesis	4 (0.04)	4 (0.04)	8 (0.07)	3 (0.03)	2 (0.02)	6 (0.05)	14 (0.11)	14 (0.14)	0.001
Dysplastic spondylolisthesis	0 (0.00)	1 (0.24)	0 (0.00)	1 (0.21)	0 (0.00)	0 (0.00)	0 (0.00)	0 (0.00)	0.38
Congenital kyphosis	1 (0.18)	0 (0.00)	1 (0.19)	1 (0.22)	0 (0.00)	0 (0.00)	1 (0.15)	0 (0.00)	0.50
Scheuermann’s kyphosis	0 (0.00)	1 (0.12)	0 (0.00)	0 (0.00)	0 (0.00)	0 (0.00)	1 (0.13)	1 (0.17)	0.31
Other kyphosis	18 (0.46)	17 (0.44)	21 (0.54)	10 (0.27)	13 (0.31)	15 (0.35)	9 (0.22)	6 (0.17)	0.004
Total	48 (0.10)	55 (0.12)	68 (0.14)	42 (0.10)	48 (0.10)	44 (0.09)	54 (0.11)	51 (0.12)	0.93

**Table 3:** Neurologic deficit

	2013 *N* (%)	2014 *N* (%)	2015 *N* (%)	2016 N (%)	2017 *N* (%)	2018 *N* (%)	2019 *N* (%)	2020 *N* (%)	*p* value
IS < 10	3 (0.17)	1 (0.05)	3 (0.17)	3 (0.15)	3 (0.17)	2 (0.12)	2 (0.12)	5 (0.39)	0.27
IS 10–18	37 (0.37)	48 (0.50)	31 (0.29)	36 (0.38)	37 (0.36)	39 (0.38)	36 (0.36)	27 (0.31)	0.28
IS > 18	38 (0.90)	30 (0.85)	30 (0.77)	29 (0.91)	40 (0.98)	34 (0.97)	28 (0.76)	27 (0.78)	0.73
Congenital scoliosis	33 (1.24)	28 (1.21)	25 (1.03)	20 (0.88)	36 (1.47)	29 (1.15)	36 (1.59)	21 (1.12)	0.49
Neuromuscular scoliosis	28 (0.74)	24 (0.65)	19 (0.50)	12 (0.35)	22 (0.57)	17 (0.45)	17 (0.51)	9 (0.32)	0.03
Other scoliosis	77 (1.30)	96 (1.66)	81 (1.40)	49 (0.97)	78 (1.38)	68 (1.11)	64 (1.06)	41 (0.89)	0.001
Isthmic spondylolisthesis	26 (0.67)	35 (1.06)	31 (0.85)	27 (0.83)	20 (0.56)	28 (0.75)	25 (0.65)	23 (0.74)	0.31
Degenerative spondylolisthesis	59 (0.53)	71 (0.70)	78 (0.68)	57 (0.57)	60 (0.52)	74 (0.62)	69 (0.57)	52 (0.54)	0.42
Dysplastic spondylolisthesis	19 (3.36)	11 (2.63)	23 (4.76)	15 (3.10)	13 (2.53)	7 (1.20)	15 (2.80)	9 (2.30)	0.06
Congenital kyphosis	13 (2.32)	13 (3.07)	15 (2.80)	18 (4.03)	13 (2.65)	15 (2.65)	7 (1.08)	13 (3.10)	0.38
Scheuermann’s kyphosis	4 (0.44)	6 (0.74)	4 (0.44)	3 (0.47)	3 (0.41)	4 (0.58)	1 (0.13)	3 (0.50)	0.46
Other kyphosis	54 (0.46)	67 (1.73)	67 (1.73)	74 (1.99)	61 (1.47)	76 (1.77)	61 (1.51)	57 (1.65)	0.86
Total	391 (0.80)	430 (0.94)	407 (0.83)	343 (0.78)	386 (0.78)	393 (0.79)	361 (0.74)	287 (0.71)	0.002

**Table 4:** Infection

	2013 2 *N* (%)	2014 3 *N* (%)	2015 4 *N* (%)	2016 5 *N* (%)	2017 6 *N* (%)	2018 7 *N* (%)	2019 8 *N* (%)	2020 9 *N* (%)	*p* value
IS < 10	16 (0.91)	11 (0.64)	16 (0.89)	7 (0.36)	16 (0.90)	6 (0.36)	5 (0.31)	5 (0.39)	0.01
IS 10–18	53 (0.54)	66 (0.69)	64 (0.61)	56 (0.59)	55 (0.53)	44 (0.43)	53 (0.53)	40 (0.46)	0.05
IS > 18	43 (1.02)	44 (1.25)	41 (1.05)	32 (1.00)	43 (1.06)	35 (0.99)	32 (0.87)	33 (0.95)	0.26
Congenital scoliosis	20 (0.75)	12 (0.52)	28 (1.16)	23 (1.01)	15 (0.61)	15 (0.60)	25 (1.10)	14 (0.74)	0.74
Neuromuscular scoliosis	142 (3.73)	119 (3.24)	151 (3.94)	117 (3.44)	135 (3.47)	133 (3.54)	122 (3.68)	86 (3.02)	0.39
Other scoliosis	92 (1.55)	84 (1.45)	87 (1.50)	60 (1.19)	76 (1.34)	75 (1.23)	88 (1.46)	53 (1.15)	0.10
Isthmic spondylolisthesis	20 (0.51)	33 (1.00)	30 (0.82)	17 (0.52)	12 (0.33)	22 (0.88)	11 (0.29)	17 (0.55)	0.01
Degenerative spondylolisthesis	115 (1.04)	112 (1.10)	125 (1.08)	109 (1.10)	96 (0.83)	103 (0.86)	101 (0.84)	72 (0.74)	> 0.001
Dysplastic spondylolisthesis	1 (0.18)	4 (0.95)	0 (0.00)	1 (0.21)	2 (0.39)	3 (0.52)	4 (0.75)	2 (0.51)	0.33
Congenital kyphosis	6 (1.07)	7 (1.65)	13 (2.42)	2 (0.45)	3 (0.61)	1 (0.18)	2 (0.31)	1 (0.24)	> 0.001
Scheuermann’s kyphosis	12 (1.32)	14 (1.73)	13 (1.45)	7 (1.10)	9 (1.23)	7 (1.01)	13 (1.73)	5 (0.84)	0.46
Other kyphosis	66 (1.69)	73 (1.24)	72 (1.86)	54 (1.45)	72 (1.73)	46 (1.07)	68 (1.69)	58 (1.68)	0.21
Total	586 (1.19)	579 (1.27)	640 (1.30)	485 (1.11)	534 (1.08)	490 (0.99)	524 (1.08)	386 (0.95)	> 0.001

**Table 5:** Unintentional return to OR

	2017 *N* (%)	2018 *N* (%)	2019 *N* (%)	2020 *N* (%)	*p* value
IS < 10	23 (1.29)	20 (1.19)	10 (0.62)	8 (0.62)	0.02
IS 10–18	93 (0.90)	81 (0.79)	84 (0.84)	71 (0.82)	0.66
IS > 18	58 (1.06)	57 (1.62)	48 (1.30)	54 (1.55)	0.93
Congenital scoliosis	40 (1.64)	36 (1.02)	47 (2.07)	33 (1.76)	0.40
Neuromuscular scoliosis	150 (3.86)	135 (3.60)	132 (3.99)	90 (3.16)	0.30
Other scoliosis	159 (2.81)	152 (2.50)	146 (2.42)	105 (2.28)	0.09
Isthmic spondylolisthesis	32 (0.90)	33 (0.88)	31 (0.81)	30 (0.98)	0.86
Degenerative spondylolisthesis	149 (1.28)	125 (1.04)	167 (1.39)	125 (1.29)	0.43
Dysplastic spondylolisthesis	12 (2.33)	7 (1.20)	10 (1.87)	2 (0.51)	0.08
Congenital kyphosis	13 (2.65)	12 (2.12)	10 (1.55)	9 (2.14)	0.42
Scheuermann’s kyphosis	18 (2.47)	12 (1.74)	16 (2.13)	11 (1.84)	0.54
Other kyphosis	175 (4.21)	122 (2.84)	109 (2.71)	115 (3.34)	0.02
Total	882 (1.79)	792 (1.60)	810 (1.66)	653 (1.61)	0.086

**Table 6:** Vision loss

	2013 *N* (%)	2014 *N* (%)	2015 *N* (%)	*p* value
IS < 10	0 (0.00)	0 (0.00)	0 (0.00)	–
IS 10–18	0 (0.00)	0 (0.00)	2 (0.02)	1
IS > 18	1 (0.02)	2 (0.06)	2 (0.05)	0.55
Congenital scoliosis	0 (0.00)	1 (0.04)	0 (0.00)	0.97
Neuromuscular scoliosis	0 (0.00)	0 (0.00)	0 (0.00)	–
Other scoliosis	0 (0.00)	1 (0.02)	3 (0.05)	0.12
Isthmic spondylolisthesis	0 (0.00)	0 (0.00)	1 (0.03)	1
Degenerative spondylolisthesis	1 (< 0.01)	0 (0.00)	4 (0.03)	0.15
Dysplastic spondylolisthesis	0 (0.00)	0 (0.00)	0 (0.00)	–
Congenital kyphosis	1 (0.18)	0 (0.00)	0 (0.00)	1
Scheuermann’s kyphosis	0 (0.00)	2 (0.25)	0 (0.00)	–
Other kyphosis	1 (0.03)	0(0.00)	1 (0.03)	0.99
Total	4 (< 0.01)	6 (< 0.01)	13 (0.03)	0.03

The overall infection rate varied from year to year as well. The year with the lowest infection rate was 2020 at 0.95% and the year with the highest infection rate was 2015 at 1.30%. The NM group had the highest infection rate, ranging from 3.24 to 3.94%. These data are in accordance with the study published by Burton et al., who reported an overall infection rate of 1.14% in 2012, with the NM scoliosis group having the highest infection rate at 2.97% [4].

The overall neurological deficit rate ranged from 0.71 to 0.94%, with congenital kyphosis and dysplastic spondylolisthesis patients having the highest rates. These rates appear to be slightly greater than the previously published data from 2009 to 2012 (0.44–0.79%) but the groups that had the highest rates of neurological deficits remained unchanged.

As stated above, vision loss rates were not recorded after 2015. However, from 2013 to 2015, visual loss rates were less than 0.1% in all groups, similar to previously published accounts. There were 13 cases of blindness reported in 2015 for a rate of 0.03%.

Data on unintentional return to the OR were first collected in 2017 during which the reported rate was 1.79%. This remained similar in the two subsequent years with reported unintentional return to the OR rates at 1.60% in 2018, 1.66% in 2019 and 1.61% in 2020.

Multiple groups were found to have a statistically significant decrease in complication rate from 2013 to 2020 (Supplement). In terms of infection, the groups that were found to have a statistically significant decreasing trend were the IS < 10 (*p* = 0.01), IS 10–18 (*p* = 0.05), isthmic spondylolisthesis (*p* = 0.01), degenerative spondylolisthesis (*p* < 0.01), and congenital kyphosis (*p* < 0.01) groups. Furthermore, there was an overall statistically significant decreasing trend in infection across all cases (*p* < 0.01). In addition, the neuromuscular scoliosis group (p = 0.03), other scoliosis group (*p* < 0.01), and all cases (*p* < 0.01) showed a statistically significant decreasing trend in neurologic deficit. Unintentional return to the OR was found to significantly decrease in the IS < 10 group (*p* = 0.02) and the other kyphosis group (*p* = 0.02). Deaths were found to significantly decrease in the other kyphosis group (*p* < 0.01). The only statistically significant increasing trends were vision loss across all cases (*p* = 0.03) and deaths in the degenerative spondylolisthesis group (*p* < 0.01).

## Discussion

Burton et al. were the first to report on the SRS M&M database after it was changed to include more specific reporting of four major complication categories (death, visual deficit, neurological deficit, infection) starting in 2009 [4]. As shown by our data, these complication rates have remained relatively constant over the past decade.

Patients with NM scoliosis had the highest rate of infection throughout the years. This is consistent with the previous report by Burton et al. [4]. The data presented include for both superficial and deep surgical site infections. Mackenzie et al. reported on the surgical site infection rate in patients undergoing instrumentation for scoliosis and found that patients with NM scoliosis had the highest infection rate compared to other scoliosis etiologies as well [12]. Their study showed a higher rate of 13.1% but their patients were followed out to 1 year postoperatively, whereas the SRS database only captures acute infections that occur within 90 days of surgery. Previous studies have noted that the overall risk for surgical site infection in AIS patients ranges from 0.5 to 1.6%, which is consistent with our findings [13].

As with the previous study by Burton et al., congenital kyphosis and dysplastic spondylolisthesis patients had the highest neurological deficit rates [4]. These rates fluctuated from year to year with no clear trend. Several series on the surgical treatment of congenital kyphosis have reported postoperative neurological deficit as a complication. In their group of 23 patients treated with either pedicle subtraction osteotomy or vertebral column resection, Zeng et al. had two (8.7%) patients with a neurological deficit [14]. Both of these patients recovered. Kim et al. reported similar results, in which 2 out of 26 (7.6%) patients had a neurological deficit after treatment for congenital kyphosis [15]. Molinari et al. analyzed 32 patients treated surgically for Meyerding Grade 3 or 4 isthmic dysplastic spondylolisthesis and found only one (3.1%) patient with a neurological deficit who eventually recovered [16].

Similar to the study by Burton et al., the overall mortality rate remained low [4]. Whereas Burton et al. found that the NM scoliosis group had the highest mortality rate, in the time period from 2013 to 2020, NM patients had the second highest mortality rate behind patients in the other kyphosis group. There were several years in which NM patients had a higher mortality rate than the other kyphosis group (2016–2017, 2019–2020). In their prospectively collected, multicenter database in cerebral palsy patients treated surgically, Yaszay et al. found that 11 of 257 (4.3%) patients died between 3 months and 5.6 years postoperatively [17]. In addition, 4 (1.5%) patients died less than a year after surgery and 2 (0.7%) deaths were directly related to the surgical procedure.

In 2017, data for unintentional return to the OR began to be collected. The overall rate was found to be 1.66%. Several studies have looked at unplanned returns to the operating room after spinal surgery. Samdani et al. found a 2.0% unplanned return rate in AIS patients who underwent pedicle screw fixation [18]. In their study, Mehta et al. found an unplanned return to the operating room rate of 4.8% in AIS [19]. Their average time to return to the OR was approximately 734 days.

Infection appeared to decrease significantly year over year in certain groups. This could be attributed to the increased use of vancomycin powder at wound closure. Two groups (neuromuscular scoliosis and other scoliosis) showed a significantly decreasing trend in neurologic deficit. Interestingly, Burton et al. showed an increasing trend in neurologic deficits from 2009 to 2012. The other kyphosis group trended significantly downward for deaths and unintentional return to the OR. The only group to demonstrate a significantly increasing trend was the degenerative spondylolisthesis group with death rate.

Although there was an increase in vision loss cases, these data have not been captured in the M&M database since 2015. This was due to blindness being such a rare event (1 out of 3000 cases). Despite the rarity, it is a devastating consequence and surgeons should be acutely aware of head positioning and avoiding pressure on the eyes when positioning a patient prior to surgery.

It is unclear as to why the mortality rate for degenerative spondylolisthesis increased during the reporting period. Since 2017, the number of cases reported for degenerative spondylolisthesis have increased except for 2020 most likely due to the COVID-19 pandemic. It is possible the increase in cases has resulted in patients with more medical comorbidities undergoing surgery thus resulting in a higher death rate.

This study has several drawbacks, including its retrospective nature, and it is purely descriptive in nature with no p value corrections despite concurrent analyses. In addition, this study relies on members to accurately and completely report their data. Reporting is voluntary and there is no quality assurance process, possibly resulting in a lower rate of complications if the data is incomplete. We also recognize that medical complications are not recorded in our database so the complication rate reported here is most likely much lower than the total complication rate for these procedures. We advise that surgeon performance should not be compared to these data as well as there was no risk adjustment performed. The advantages of this study include the large number of cases. The number of surgeons reporting cases have a wide range of experience and the large number of institutions represented from around the world may help control for differences in patient populations.

## Conclusion

The reported complication rates in our study are comparable to years past. There was a decreasing overall trend for infection and neurological deficit. Patients with NM scoliosis had the highest rate of infection. Congenital kyphosis and dysplastic spondylolisthesis patients had the highest neurological deficit rates. While the other kyphosis group had the highest mortality rate, there was a decreasing trend in mortality rate over time. The only increasing trend was mortality rate in the degenerative spondylolisthesis group. Unintentional return to the OR ranged from 1.60 to 1.79%. Given the large number of patients included in the registry, the data presented here should give spine surgeons guidance when counseling patients on complications after surgery.

## Supplementary Information

Below is the link to the electronic supplementary material.

Supplementary file1 Fig.1 Statistically significant trend in IS < 10 infection (DOCX 16 kb)

Supplementary file2 Fig.2 Statistically significant trend in IS 10-18 infection (DOCX 16 kb)

Supplementary file3 Fig.3 Statistically significant trend in isthmic spondylolisthesis infection (DOCX 16 kb)

Supplementary file4 Fig.4 Statistically significant trend in degenerative spondylolisthesis infection (DOCX 15 kb)

Supplementary file5 Fig.5 Statistically significant trend in congenital kyphosis infection (DOCX 15 kb)

Supplementary file6 Fig.6 Statistically significant trend in all cases infection (DOCX 15 kb)

Supplementary file7 Fig.7 Statistically significant trend in neuromuscular scoliosis neurologic deficit (DOCX 15 kb)

Supplementary file8 Fig.8 Statistically significant trend in other scoliosis neurological deficit (DOCX 15 kb)

Supplementary file9 Fig.9 Statistically significant trend in all cases neurological deficit (DOCX 15 kb)

Supplementary file10 Fig.10 Statistically significant trend in IS < 10 unintentional return to OR (DOCX 15 kb)

Supplementary file11 Fig.11 Statistically significant trend in other kyphosis unintentional return to OR (DOCX 15 kb)

Supplementary file12 Fig.12 Statistically significant trend in degenerative spondylolisthesis deaths (DOCX 15 kb)

Supplementary file13 Fig.13 Statistically significant trend in other kyphosis deaths (DOCX 15 kb)

Supplementary file14 Fig.14 Statistically significant trend in all cases vision loss (DOCX 15 kb)

## Data Availability

The datasets used and/or analyzed during the current study are available from the corresponding author on reasonable request.

## Code availability

The software application and code used for the statistical analysis are available from the corresponding author on reasonable request.
