# Supplementary material for: Spinal deformity surgery is accompanied by serious complications: report from the Morbidity and Mortality Database of the Scoliosis Research Society from 2013 to 2020
Source: Spine Deform. 2022 Jul 15;10(6):1307–13. doi: 10.1007/s43390-022-00548-y (PMC9284960; doi:10.1007/s43390-022-00548-y)
Supplement: Supplementary file 12 — Supplementary file12 Fig.12 Statistically significant trend in degenerative spondylolisthesis deaths (DOCX 15 kb) [file 43390_2022_548_MOESM12_ESM.docx]

Fig12.
